# (η^3^-All­yl)bromido(1-phenyl-1*H*-imidazole-κ*N* ^3^)palladium(II)

**DOI:** 10.1107/S1600536811008798

**Published:** 2011-03-15

**Authors:** Jiangying Huang, Xiao Zhang

**Affiliations:** a College of Food Science and Biotechnology, Zhejiang Gongshang University, Hangzhou 310035, People’s Republic of China

## Abstract

The title compound, [PdBr(C_3_H_5_)(C_9_H_8_N_2_)], was synthesized by the reaction of the allyl­palladium(II) bromide dimer and 1-phenyl-1*H*-imidazole. The Pd atom is coordinated by one allyl group [in η^3^ mode, the central CH group of the allyl group is disordered over two sets of sites in a 0.668 (5):0.332 (5) ratio], one bromide anion and a 1-phenyl-1*H*-imidazole ligand. Intra­molecular face-to-face π–π stacking inter­actions occur between adjacent phenyl or imidazole groups, with centroid–centroid distances in the range 3.877 (1)–3.6596 (6) Å, forming a supra­molecular chain along [100].

## Related literature

For applications of allyl­palladium(II) complexes in catalysis, see: Amatore *et al.* (2005 ▶); Faller & Sarantopoulos (2004 ▶); Johannsen & Jørgensen (1998 ▶); Li *et al.* (2006 ▶); Trost & Van Vranken (1996 ▶); Viciu *et al.* (2002 ▶). For the crystal structure of a 1-phenyl-1*H*-imidazole derivative, see: Huynh & Wu (2009 ▶).
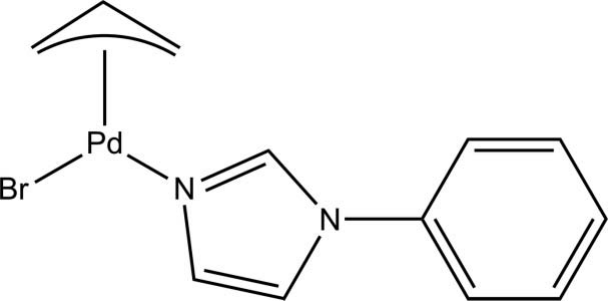


## Experimental

### 

#### Crystal data

[PdBr(C_3_H_5_)(C_9_H_8_N_2_)]
*M* *_r_* = 371.55
Monoclinic, 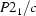

*a* = 9.813 (2) Å
*b* = 9.5376 (19) Å
*c* = 13.534 (3) Å
β = 92.30 (3)°
*V* = 1265.6 (4) Å^3^
*Z* = 4
Mo *K*α radiation
μ = 4.60 mm^−1^
*T* = 293 K
0.29 × 0.20 × 0.10 mm

#### Data collection

Rigaku R-AXIS RAPID diffractometer
Absorption correction: multi-scan (*ABSCOR*; Higashi, 1995 ▶) *T*_min_ = 0.345, *T*_max_ = 0.631
12155 measured reflections
2904 independent reflections
2612 reflections with *I* > 2σ(*I*)
*R*_int_ = 0.023

#### Refinement

*R*[*F* ^2^ > 2σ(*F* ^2^)] = 0.021
*wR*(*F* ^2^) = 0.049
*S* = 1.05
2904 reflections
150 parameters
H-atom parameters constrained
Δρ_max_ = 0.41 e Å^−3^
Δρ_min_ = −0.42 e Å^−3^

### 

Data collection: *RAPID-AUTO* (Rigaku, 1998 ▶); cell refinement: *RAPID-AUTO*; data reduction: *CrystalStructure* (Rigaku/MSC, 2004) ▶; program(s) used to solve structure: *SHELXS97* (Sheldrick, 2008 ▶); program(s) used to refine structure: *SHELXL97* (Sheldrick, 2008 ▶); molecular graphics: *ORTEPII* (Johnson, 1976 ▶); software used to prepare material for publication: *SHELXL97*.

## Supplementary Material

Crystal structure: contains datablocks I, global. DOI: 10.1107/S1600536811008798/rn2079sup1.cif

Structure factors: contains datablocks I. DOI: 10.1107/S1600536811008798/rn2079Isup2.hkl

Additional supplementary materials:  crystallographic information; 3D view; checkCIF report

## Figures and Tables

**Fig. 1.**
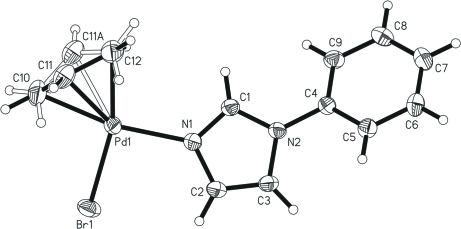
ORTEP view of the title compound. The displacement ellipsoids are drawn at 30% probability level, (the minor disordered component is shown as well as the major).

**Fig. 2.**
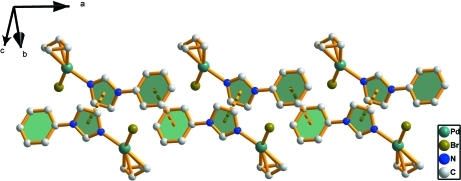
The one-dimensional chain of the compound (dashed lines represent π-π stacking interactions, H atoms are omitted for clarity).

## supplementary crystallographic information

### Comment

Allylpalladium(II) complexes are used as catalysts in various organic syntheses such as palladium-mediated coupling reaction, allylic alkylation, allylic amination, and other allylic substitutions (Trost & Van Vranken, 1996; Johannsen & Jørgensen, 1998; Viciu *et al.*, 2002). The crystallographic structures of allylpalladium complexes with a variety of ligands have been studied to elucidate the mechanism of palladium-catalyzed allylic substitution (Faller & Sarantopoulos, 2004; Amatore *et al.*, 2005; Li *et al.*, 2006). Herein, we report the crystal structure of a allylpalladium(II) complex, with 1-phenyl-1*H*-imidazole as the supporting ligand.

In the crystal structure, each palladium atom is coordinated to one allyl group (in η^3^ mode), one bromine anion and a 1-phenyl-1*H*-imidazole group, the bond lengths and angles have normal values (Huynh & Wu, 2009). Intramolecular face-to-face π-stacking interactions exist between adjacent phenyl or imidazolene groups, with centroid-centroid distances of 3.877 (1)–3.6596 (6) Å, forming a supramolecular chain along the [100] direction.

### Experimental

A solution of allylpalladium(II) bromide dimer (0.227 g, 0.500 mmol) and 1-phenyl-1*H*-imidazole (0.144 g, 1.00 mmol) in THF (5 ml) was stirred for 5 h at room temperature under a nitrogen atmosphere. The mixture was then filtered over celite and the solid was washed with THF (2 τimes 5 ml). The filtrate was evaporated on a rotary evaporator and the residue was purified by flash chromatography on silica gel with ethyl acetate/hexane (1:1) to give the title compound as a white solid (0.256 g, 69%). Colorless crystals were obtained by vapor diffusion of hexane into an ethyl acetate solution over a period of 7 d.

### Refinement

H atoms were positioned geometrically and treated as riding atoms (C—H = 0.93–0.97 Å, with *U*_iso_(H) = 1.2 *U*_eq_(C)). The central CH (C11,H11) of the allyl group is disordered; the occupancies of the two sites were 0.67 (C11, H11) and 0.33 (C11A, H11A). The displacement parameters of C11 and C11*a* were constrained to be the same using the EADP constraint.

### Crystal data

**Table:**

[PdBr(C_3_H_5_)(C_9_H_8_N_2_)]	*F*(000) = 720
*M**_r_* = 371.55	*D*_x_ = 1.950 Mg m^−^^3^
Monoclinic, *P*2_1_/*c*	Mo *K*α radiation, λ = 0.71073 Å
Hall symbol: -P 2ybc	Cell parameters from 10721 reflections
*a* = 9.813 (2) Å	θ = 3.0–27.5°
*b* = 9.5376 (19) Å	µ = 4.60 mm^−^^1^
*c* = 13.534 (3) Å	*T* = 293 K
β = 92.30 (3)°	Block, yellow
*V* = 1265.6 (4) Å^3^	0.29 × 0.20 × 0.10 mm
*Z* = 4	

### Data collection

**Table:**

Rigaku R-AXIS RAPID diffractometer	2904 independent reflections
Radiation source: fine-focus sealed tube	2612 reflections with *I* > 2σ(*I*)
graphite	*R*_int_ = 0.023
Detector resolution: 0 pixels mm^-1^	θ_max_ = 27.5°, θ_min_ = 3.0°
ω scans	*h* = −12→12
Absorption correction: multi-scan (*ABSCOR*; Higashi, 1995)	*k* = −10→12
*T*_min_ = 0.345, *T*_max_ = 0.631	*l* = −17→17
12155 measured reflections	

### Refinement

**Table:**

Refinement on *F*^2^	Secondary atom site location: difference Fourier map
Least-squares matrix: full	Hydrogen site location: inferred from neighbouring sites
*R*[*F*^2^ > 2σ(*F*^2^)] = 0.021	H-atom parameters constrained
*wR*(*F*^2^) = 0.049	*w* = 1/[σ^2^(*F*_o_^2^) + (0.0207*P*)^2^ + 0.7032*P*] where *P* = (*F*_o_^2^ + 2*F*_c_^2^)/3
*S* = 1.05	(Δ/σ)_max_ = 0.002
2904 reflections	Δρ_max_ = 0.41 e Å^−^^3^
150 parameters	Δρ_min_ = −0.42 e Å^−^^3^
0 restraints	Extinction correction: *SHELXL97* (Sheldrick, 2008), Fc^*^=kFc[1+0.001xFc^2^λ^3^/sin(2θ)]^-1/4^
Primary atom site location: structure-invariant direct methods	Extinction coefficient: 0.0052 (3)

### Special details

**Table:**

Geometry. All e.s.d.'s (except the e.s.d. in the dihedral angle between two l.s. planes) are estimated using the full covariance matrix. The cell e.s.d.'s are taken into account individually in the estimation of e.s.d.'s in distances, angles and torsion angles; correlations between e.s.d.'s in cell parameters are only used when they are defined by crystal symmetry. An approximate (isotropic) treatment of cell e.s.d.'s is used for estimating e.s.d.'s involving l.s. planes.
Refinement. Refinement of *F*^2^ against ALL reflections. The weighted *R*-factor *wR* and goodness of fit *S* are based on *F*^2^, conventional *R*-factors *R* are based on *F*, with *F* set to zero for negative *F*^2^. The threshold expression of *F*^2^ > σ(*F*^2^) is used only for calculating *R*-factors(gt) *etc*. and is not relevant to the choice of reflections for refinement. *R*-factors based on *F*^2^ are statistically about twice as large as those based on *F*, and *R*- factors based on ALL data will be even larger.

### Fractional atomic coordinates and isotropic or equivalent isotropic displacement parameters (Å^2^)

**Table:**

	*x*	*y*	*z*	*U*_iso_*/*U*_eq_	Occ. (<1)
Pd1	0.838252 (17)	0.099243 (18)	0.493735 (12)	0.03674 (7)	
Br1	0.82502 (3)	0.05502 (3)	0.675655 (18)	0.05630 (9)	
N1	0.99538 (19)	0.24930 (19)	0.50757 (13)	0.0387 (4)	
N2	1.17421 (19)	0.37658 (18)	0.47351 (13)	0.0360 (4)	
C1	1.0840 (2)	0.2791 (2)	0.44025 (16)	0.0383 (5)	
H1	1.0843	0.2383	0.3779	0.046*	
C2	1.0296 (2)	0.3323 (3)	0.58786 (17)	0.0440 (5)	
H2	0.9843	0.3334	0.6469	0.053*	
C3	1.1386 (2)	0.4120 (3)	0.56818 (17)	0.0422 (5)	
H3	1.1813	0.4775	0.6098	0.051*	
C4	1.2881 (2)	0.4302 (2)	0.42216 (16)	0.0359 (5)	
C5	1.3264 (2)	0.5684 (2)	0.43619 (18)	0.0420 (5)	
H5	1.2777	0.6264	0.4773	0.050*	
C6	1.4379 (3)	0.6194 (3)	0.3884 (2)	0.0491 (6)	
H6	1.4646	0.7122	0.3976	0.059*	
C7	1.5097 (3)	0.5334 (3)	0.32725 (18)	0.0500 (6)	
H7	1.5848	0.5682	0.2954	0.060*	
C8	1.4703 (3)	0.3963 (3)	0.31338 (18)	0.0486 (6)	
H8	1.5185	0.3389	0.2716	0.058*	
C9	1.3595 (2)	0.3432 (3)	0.36110 (17)	0.0439 (5)	
H9	1.3334	0.2501	0.3522	0.053*	
C10	0.6835 (3)	−0.0427 (3)	0.4455 (2)	0.0635 (8)	
H1AA	0.7624	−0.0937	0.4606	0.076*	0.668 (5)
H10	0.6024	−0.0662	0.4747	0.076*	0.668 (5)
H1BC	0.6510	0.0322	0.4817	0.076*	0.332 (5)
H10A	0.6601	−0.1340	0.4620	0.076*	0.332 (5)
C11	0.6866 (4)	0.0633 (4)	0.3823 (3)	0.0560 (9)	0.668 (5)
H11	0.6082	0.1269	0.3775	0.067*	0.668 (5)
C11A	0.7676 (9)	−0.0181 (9)	0.3661 (6)	0.0560 (9)	0.332 (5)
H11A	0.8256	−0.0970	0.3482	0.067*	0.332 (5)
C12	0.8065 (3)	0.1021 (3)	0.3366 (2)	0.0634 (8)	
H1AB	0.8870	0.0531	0.3503	0.076*	0.668 (5)
H12	0.8057	0.1770	0.2925	0.076*	0.668 (5)
H1BD	0.7788	0.1829	0.3686	0.076*	0.332 (5)
H12A	0.8627	0.1089	0.2830	0.076*	0.332 (5)

### Atomic displacement parameters (Å^2^)

**Table:**

	*U*^11^	*U*^22^	*U*^33^	*U*^12^	*U*^13^	*U*^23^
Pd1	0.03762 (10)	0.03528 (10)	0.03758 (10)	0.00007 (7)	0.00452 (7)	0.00324 (7)
Br1	0.0825 (2)	0.04785 (16)	0.04009 (14)	−0.00322 (13)	0.02166 (13)	0.00146 (10)
N1	0.0414 (10)	0.0383 (10)	0.0367 (9)	−0.0019 (8)	0.0052 (8)	−0.0019 (8)
N2	0.0390 (10)	0.0340 (9)	0.0354 (9)	−0.0002 (7)	0.0068 (7)	−0.0038 (7)
C1	0.0442 (12)	0.0351 (11)	0.0358 (11)	−0.0014 (9)	0.0048 (9)	−0.0039 (9)
C2	0.0460 (13)	0.0519 (14)	0.0345 (11)	−0.0005 (11)	0.0078 (10)	−0.0060 (10)
C3	0.0451 (13)	0.0468 (13)	0.0349 (11)	0.0004 (10)	0.0034 (9)	−0.0094 (9)
C4	0.0348 (11)	0.0361 (11)	0.0369 (11)	0.0015 (8)	0.0021 (9)	0.0011 (9)
C5	0.0404 (12)	0.0383 (12)	0.0473 (13)	0.0026 (9)	0.0017 (10)	−0.0022 (10)
C6	0.0459 (14)	0.0423 (13)	0.0589 (15)	−0.0083 (10)	−0.0020 (12)	0.0070 (11)
C7	0.0379 (13)	0.0650 (17)	0.0472 (14)	−0.0030 (11)	0.0025 (10)	0.0148 (12)
C8	0.0466 (14)	0.0578 (15)	0.0422 (13)	0.0110 (11)	0.0098 (10)	0.0034 (11)
C9	0.0503 (14)	0.0389 (12)	0.0428 (12)	0.0043 (10)	0.0058 (10)	−0.0029 (10)
C10	0.0516 (16)	0.0663 (18)	0.0718 (19)	−0.0195 (14)	−0.0062 (14)	0.0046 (15)
C11	0.051 (2)	0.055 (2)	0.060 (2)	0.0001 (15)	−0.0210 (17)	0.0009 (18)
C11A	0.051 (2)	0.055 (2)	0.060 (2)	0.0001 (15)	−0.0210 (17)	0.0009 (18)
C12	0.0651 (18)	0.082 (2)	0.0424 (14)	−0.0131 (15)	−0.0107 (13)	0.0077 (13)

### Geometric parameters (Å, °)

**Table:**

Pd1—C11	2.104 (4)	C6—H6	0.9300
Pd1—N1	2.1066 (19)	C7—C8	1.374 (4)
Pd1—C10	2.118 (3)	C7—H7	0.9300
Pd1—C12	2.138 (3)	C8—C9	1.383 (3)
Pd1—C11A	2.149 (8)	C8—H8	0.9300
Pd1—Br1	2.5064 (6)	C9—H9	0.9300
N1—C1	1.315 (3)	C10—C11	1.326 (5)
N1—C2	1.375 (3)	C10—C11A	1.400 (10)
N2—C1	1.349 (3)	C10—H1AA	0.9300
N2—C3	1.383 (3)	C10—H10	0.9300
N2—C4	1.434 (3)	C10—H1BC	0.9300
C1—H1	0.9300	C10—H10A	0.9300
C2—C3	1.347 (3)	C11—C12	1.402 (5)
C2—H2	0.9300	C11—H11	0.9800
C3—H3	0.9300	C11A—C12	1.278 (9)
C4—C9	1.381 (3)	C11A—H11A	0.9800
C4—C5	1.382 (3)	C12—H1AB	0.9300
C5—C6	1.381 (3)	C12—H12	0.9300
C5—H5	0.9300	C12—H1BD	0.9300
C6—C7	1.380 (4)	C12—H12A	0.9300
			
C11—Pd1—N1	131.96 (13)	C11—C10—H1AA	120.0
C11—Pd1—C10	36.59 (14)	C11A—C10—H1AA	75.2
N1—Pd1—C10	167.14 (10)	Pd1—C10—H1AA	71.7
C11—Pd1—C12	38.59 (15)	C11—C10—H10	120.0
N1—Pd1—C12	99.00 (10)	C11A—C10—H10	155.1
C10—Pd1—C12	68.14 (12)	Pd1—C10—H10	129.6
C11—Pd1—C11A	31.0 (3)	H1AA—C10—H10	120.0
N1—Pd1—C11A	129.5 (3)	C11—C10—H1BC	76.7
C10—Pd1—C11A	38.3 (3)	C11A—C10—H1BC	120.0
C12—Pd1—C11A	34.7 (2)	Pd1—C10—H1BC	66.6
C11—Pd1—Br1	127.85 (13)	H1AA—C10—H1BC	125.9
N1—Pd1—Br1	95.37 (5)	H10—C10—H1BC	69.1
C10—Pd1—Br1	97.49 (9)	C11—C10—H10A	151.8
C12—Pd1—Br1	165.57 (8)	C11A—C10—H10A	120.0
C11A—Pd1—Br1	132.4 (2)	Pd1—C10—H10A	134.8
C1—N1—C2	106.00 (19)	H1AA—C10—H10A	70.7
C1—N1—Pd1	125.94 (15)	H10—C10—H10A	56.6
C2—N1—Pd1	128.04 (15)	H1BC—C10—H10A	120.0
C1—N2—C3	106.88 (19)	C10—C11—C12	121.9 (4)
C1—N2—C4	126.74 (19)	C10—C11—Pd1	72.3 (2)
C3—N2—C4	126.35 (19)	C12—C11—Pd1	72.0 (2)
N1—C1—N2	111.17 (19)	C10—C11—H11	118.5
N1—C1—H1	124.4	C12—C11—H11	118.5
N2—C1—H1	124.4	Pd1—C11—H11	118.5
C3—C2—N1	109.8 (2)	C12—C11A—C10	125.7 (8)
C3—C2—H2	125.1	C12—C11A—Pd1	72.2 (4)
N1—C2—H2	125.1	C10—C11A—Pd1	69.6 (4)
C2—C3—N2	106.1 (2)	C12—C11A—H11A	115.3
C2—C3—H3	126.9	C10—C11A—H11A	115.3
N2—C3—H3	126.9	Pd1—C11A—H11A	115.3
C9—C4—C5	120.9 (2)	C11A—C12—C11	50.0 (4)
C9—C4—N2	120.0 (2)	C11A—C12—Pd1	73.1 (4)
C5—C4—N2	119.11 (19)	C11—C12—Pd1	69.4 (2)
C6—C5—C4	119.2 (2)	C11A—C12—H1AB	75.4
C6—C5—H5	120.4	C11—C12—H1AB	120.0
C4—C5—H5	120.4	Pd1—C12—H1AB	72.9
C7—C6—C5	120.3 (2)	C11A—C12—H12	153.4
C7—C6—H6	119.8	C11—C12—H12	120.0
C5—C6—H6	119.8	Pd1—C12—H12	130.2
C8—C7—C6	120.0 (2)	H1AB—C12—H12	120.0
C8—C7—H7	120.0	C11A—C12—H1BD	120.0
C6—C7—H7	120.0	C11—C12—H1BD	75.3
C7—C8—C9	120.5 (2)	Pd1—C12—H1BD	65.2
C7—C8—H8	119.8	H1AB—C12—H1BD	125.9
C9—C8—H8	119.8	H12—C12—H1BD	70.6
C4—C9—C8	119.1 (2)	C11A—C12—H12A	120.0
C4—C9—H9	120.5	C11—C12—H12A	153.7
C8—C9—H9	120.5	Pd1—C12—H12A	135.2
C11—C10—C11A	49.3 (4)	H1AB—C12—H12A	70.5
C11—C10—Pd1	71.1 (2)	H12—C12—H12A	55.8
C11A—C10—Pd1	72.0 (3)	H1BD—C12—H12A	120.0
